# From proteins to genes: immunoanalysis in the diagnosis of muscular dystrophies

**DOI:** 10.1186/2044-5040-1-24

**Published:** 2011-06-24

**Authors:** Rita Barresi

**Affiliations:** 1 NCG Diagnostic & Advisory Service for Rare Neuromuscular Diseases, Muscle Immunoanalysis Unit, Dental Hospital, Richardson Road, Newcastle upon Tyne, UK; 2 Newcastle University, Institute of Genetic Medicine, International Centre for Life, Central Parkway, Newcastle upon Tyne, UK

## Abstract

Muscular dystrophies are a large heterogeneous group of inherited diseases that cause progressive muscle weakness and permanent muscle damage. Very few muscular dystrophies show sufficient specific clinical features to allow a definite diagnosis. Because of the currently limited capacity to screen for numerous genes simultaneously, muscle biopsy is a time and cost-effective test for many of these disorders. Protein analysis interpreted in correlation with the clinical phenotype is a useful way of directing genetic testing in many types of muscular dystrophies. Immunohistochemistry and western blot are complementary techniques used to gather quantitative and qualitative information on the expression of proteins involved in this group of diseases. Immunoanalysis has a major diagnostic application mostly in recessive conditions where the absence of labelling for a particular protein is likely to indicate a defect in that gene. However, abnormalities in protein expression can vary from absence to very subtle reduction. It is good practice to test muscle biopsies with antibodies for several proteins simultaneously and to interpret the results in context. Indeed, there is a degree of direct or functional association between many of these proteins that is reflected by the presence of specific secondary abnormalities that are of value, especially when the diagnosis is not straightforward.

## Introduction

The term muscular dystrophies (MDs) refers to a large group of genetically inherited disorders characterised by weakness and wasting of skeletal muscle. The subclassification is based on mode of inheritance, age of onset and distribution of muscles affected. Progress made in the past 25 years has enabled the discovery of new causative genetic defects with many novel proteins involved in MD (an updated list of MDs and responsible genes can be found at http://www.musclegenetable.org). Despite remarkable advances, this work is not yet complete, and although a large number of genes have been identified, a considerable number of patients still remain undiagnosed. Although a cure for most MDs is not yet available, an accurate diagnosis is key for natural history studies and to establish priorities for medical management, therapy and genetic counselling. Physical examination to determine the distribution of symptoms, together with medical and family history is central, but often the underlying genetic defect cannot be conclusively recognised on the basis of clinical information only. Indeed, differential diagnosis has to take into account the overlap of clinical features in different forms of MD and the heterogeneity in clinical presentation for many of the genes involved. Searching for gene mutations is the diagnostic gold standard but despite the rapidly evolving sequencing technologies, the analysis of multiple genes is still costly and time consuming and classification of gene mutations as pathogenic remains considerably challenging.

A wide range of laboratory tests aid in the diagnostic process. Serum level of creatine kinase (CK) is a sensitive parameter of muscle damage. The degree of CK elevation is variable in different MDs and it can give an approximate indication of the type of disorder [1]. Electromyography enables differentiation between myopathic and neurogenic processes. Muscle magnetic resonance imaging, used to determine patterns of muscle involvement, represents a promising advance in facilitating differential diagnosis [2-4]. In this context, the analysis of the muscle biopsy plays a key role in the assessment of patients with MDs and provides useful diagnostic information to direct genetic analysis.

## Histopathology and immunoanalysis

Although none of the types of MD can be distinguished on basic muscle histology, histopathology screening enables the identification of a number of features of significance when reviewed in context with clinical information. Many of the morphological abnormalities of muscle can be recognised in haematoxylin and eosin stained sections. Features such as fibre necrosis and regeneration, fibrosis and fatty infiltration, inflammation and vacuolated fibres seen in MDs are not specific to any particular type [5].

Diagnostic capabilities greatly improve when histological and histochemical tests are complemented with protein analysis. The development of antibodies (ABs) for many of the proteins affected in MDs has enabled the design of effective immunodiagnostic protocols to direct genetic screening. Identification of protein defects relies on immunohistochemical preparations and western blot analysis. Table 1 summarises the commercial ABs available for the analysis of MDs with both techniques. Tissue preparation and handling is key to the outcome of the immunoanalysis. To avoid ice crystal artefacts, skeletal muscle prepared for immunohistochemistry should be frozen in isopentane cooled in liquid nitrogen and stored at -80°C or in liquid nitrogen. Provided that protein degradation is excluded, absence of labelling for one protein generally indicates a primary defect in the gene encoding for that protein, and reduced labelling may still be useful to suggest where to start the genetic analysis. In some cases (for example, dystrophinopathy) one protein may be abnormally expressed and others secondarily reduced. For this reason it is generally recommended to test each sample with several ABs and to interpret the results by examining all proteins concurrently. Clinical data are essential not only to interpret the findings but also to select the appropriate ABs for screening, which can vary according to the diagnosis suggested. However, testing with all of the available ABs is recommended as it may lead to the identification of primary protein defects in patients with unusual phenotypes [6,7]. Since biochemical analysis requires a significant portion of a muscle biopsy, multiplex western blot techniques have become popular in diagnostic laboratories to detect several proteins simultaneously [7]. Proteins separated by gel electrophoresis and labelled by immunoblot can be evaluated by size (molecular mass) and intensity (abundance) compared to a control. A loading control such as myosin heavy chain should be used to indicate how much 'muscle' protein is loaded in each sample and to establish an adequate baseline for protein quantification, since dystrophic muscle samples may be fibrotic or contain fat. Importantly, biopsy of end stage muscle is unlikely to provide diagnostic information due to loss of myofibres and predominance of fibrovascular and adipose tissue. However a symptomatic muscle should be selected to appreciate distinctive pathologic features. Ultrasound or MRI techniques may aid the selection of the biopsy site [4,8].

**Table 1:** Commercial antibodies for the diagnosis of muscular dystrophies.

Antigen	Host/Isotype	Clone	IHC	WB
Spectrin	Mouse IgG2b	RBC2/3D5	Y	N
Dystrophin (N term)	Mouse IgG2a	Dy10/12B2	Y	Y
Dystrophin (rod domain)	Mouse IgG2a	Dy4/6D3Y	Y	Y
Dystrophin (C-term)	Mouse IgG1	Dy8/6C5	Y	Y
Dystrophin (C-term)	Mouse IgG1	MANDRA1	Y	Y
Utrophin	Mouse IgG1	DRP3/20C5	Y	N
nNOS	Mouse IgG1	RN5	Y	N
Myotilin	Mouse IgG1	RS034	Y	N
Lamin A/C	Mouse IgG2b	636	Y*	N
Caveolin-3	Mouse IgG1	26/Caveolin 3	Y	Y
Calpain-3	Mouse IgG2b	Calp3d/2C4	N	Y
Calpain-3	Mouse IgG2a	Calp3d/12A2	N	Y
Dysferlin	Mouse IgG1	Ham1/7B6	Y	Y
Dysferlin	Mouse IgG2bκ	Ham3/17B2	Y	N
α-Sarcoglycan	Mouse IgG1	Ad1/20A6	Y	Y
β-Sarcoglycan	Mouse IgG1	b-sarc/5B1	Y	N
γ-Sarcoglycan	Mouse IgG2bκ	35DAG/21B5	Y	Y
δ-Sarcoglycan	Mouse IgG2aκ	d-sarc3/12C1	Y	N
Telethonin	Mouse IgG1	G-11	N	N
α-Dystroglycan	Mouse IgM	IIH6	Y	Y
α-Dystroglycan	Mouse IgG1	VIA4-1	Y	Y
β-Dystroglycan	Mouse IgG2a	43DAG1/8D5	Y	Y
Laminin α2 80kDa	Mouse IgG1	5H2	Y	Y
Laminin α2 300kDa	Mouse IgG1κ	Mer3/22B2	Y	N
Laminin α2 300kDa	Rat IgG1	4H8	Y	N
Laminin α5	Mouse IgG2a	4C7	Y	N
Laminin β1	Mouse IgG1κ	4E10	Y*	N
Laminin γ1	Mouse IgG1κ	2E8	Y	N
Laminin γ1	Mouse IgG2a	A5	Y	N
Collagen VI	Mouse IgG1	3C4	Y	N
Collagen VI	Rat IgG1κ	VI-26	Y	N
Perlecan	Mouse IgG2aκ	A7L6	Y	N
Emerin	Mouse IgG1	4G5	Y	N
Desmin	Mouse IgG1	D33	Y	N
αB-Crystallin	Rabbit Polyclonal	G2JF	Y	N
VCP	Mouse IgG1	18/VCP	Y	N
PTRF-cavin	Mouse IgG1	4/PTRF	N	Y
MHC class I	Mouse IgG2a	W6-32	Y	N
Neonatal Myosin	Mouse IgG1	WB-MHCn	Y	N

The list includes antibodies for the detection of primary or secondary defects and for the assessment of quality and preservation of samples.

*Immunoanalysis possible but not informative for diagnostic assessment.

IHC = immunohistochemistry; MHC = major histocompatibility complex; N = labelling not carried out routinely or antibody not tested/not suitable for test; nNOS = neuronal nitric oxide synthase; PTRF = polymerase I and transcript release factor; VCP = valosin-containing protein; WB = western blot; Y = labelling carried out routinely.

**Figure 1:**
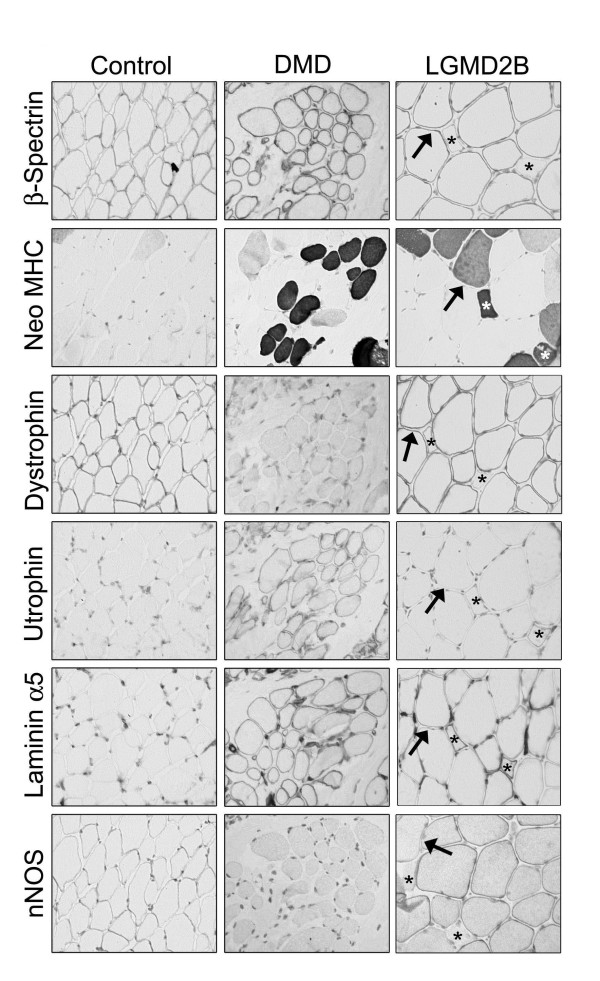
**Control tests for necrosis and regeneration**. Expression of various markers of sarcolemmal integrity (β-spectrin) and regeneration (neonatal myosin heavy chain (Neo-MHC), utrophin and laminin α5) in serial sections of control, Duchenne muscular dystrophy (DMD) and limb girdle muscular dystrophy (LGMD)2B muscle. Arrows indicate abnormal immature fibres positive for Neo-MHC; stars highlight regenerating fibres at different stages. The pattern of expression of the regeneration markers is less defined in DMD muscle due to the overall upregulation of utrophin and laminin α5 and secondary loss of neuronal nitric oxide synthase (nNOS).

The use of controls is crucial to assess the quality and preservation of samples as well as to discriminate pathological from natural changes (Table 2). In immunohistochemistry, the AB to β-spectrin serves as a marker of sarcolemmal integrity and it is used to evaluate the expression of plasma membrane proteins. In fact, necrotic fibres or fibres within degraded biopsies generally lack sarcolemmal labelling and could give false negative results. Similarly, ABs for laminin γ1 and perlecan can be used to check the integrity of the basal lamina if a reduction in laminin α2 or collagen VI is detected. By contrast, laminin β1 chain is not suitable for this purpose as it is often secondarily reduced in both recessive and dominant conditions [9]. Importantly, the expression of some proteins is developmentally regulated. For instance, β-spectrin and neuronal nitric oxide synthase (nNOS) are weakly expressed in regenerating fibres, which instead label for markers such as utrophin, desmin and major histocompatibility complex (MHC) class I [10] (Figure 1 and data not shown). Laminin α5 is also present in regenerating fibres but its expression may also be increased in dystrophinopathies and inflammatory myopathies (Figure 1). To facilitate the interpretation of these findings serial sections should also be labelled for neonatal myosin heavy chain (Neo-MHC), as a marker of regenerating fibres, but caution is needed since Neo-MHC is also found in fibres with arrested development (Figure 1) and atrophic fibres and reappears after denervation [5,11]. Other isoforms of MHC, such as embryonic MHC, are expressed more transiently in earlier stages and can be detected mainly in the smaller regenerating fibres [10].

**Table 2:** Protein controls for immunohistochemistry

Control purpose	Protein	Normal expression	Secondary changes	Notes
Preservation of plasma membrane	Spectrin	Sarcolemma	Reduction in immature/regenerating fibres. Absent or patchy in necrotic fibres. Absent of patchy in biopsies with artefacts.	May be reduced in fibres with Neo-MHC, utrophin, laminin α5, MHC class I. Absence of other sarcolemmal proteins.
Regenerating fibres	Neo-MHC	Fibres unlabelled	Labelling of regenerating fibres. Labelling of atrophic fibres.	Coexpressed with laminin α5, MHC class I, utrophin
Regenerating fibres	Utrophin	Vessels, nerves and neuromuscular junction	Labelling of regenerating fibres. Labelling of mature fibres in DMD/BMD.	Coexpressed with Neo-MHC, laminin α5, MHC class I
Regenerating fibres, denervation	nNOS	Sarcolemma	Reduction in regenerating and denervated fibres. Absent in DMD, some BMD and sarcoglycanopathies.	Reduced in fibres expressing utrophin, Neo-MHC, laminin α5, MHC class I
Regenerating fibres	Laminin α5	Blood vessels	Labelling of regenerating fibres. Labelling of mature fibres in MDC1A.	Coexpressed with utrophin, Neo-MHC, laminin α5, MHC class I
Inflammation, regenerating fibres	MHC class I	Blood vessels	Labelling of regenerating fibres. Sarcolemmal labelling in diseases with inflammatory component.	Coexpressed with utrophin, Neo-MHC, laminin α5
Basement membrane, integrity	Laminin β1	Sarcolemma and blood vessels	Sarcolemmal labelling reduced or patchy in many dominant and recessive conditions	
Basement membrane, integrity	Laminin γ1, Perlecan	Sarcolemma and blood vessels	Patchy in biopsies with artefacts	

DMD/BMD Duchenne/Becker muscular dystrophy; MDC1A = congenital muscular dystrophy type 1A; MHC = major histocompatibility complex; Neo-MHC = neonatal myosin heavy chain; nNOS = neuronal nitric oxide synthase.

## Investigation of specific disorders

Protein analysis is effective in guiding genetic analysis in many, but not all, types of MDs (Table 3). Its major diagnostic application is in recessive conditions where the protein defect can vary from absence to partial reduction. In most dominant diseases the results may be inconclusive, as both normal and abnormal alleles produce protein. Also, genetic analysis is very reliable in conditions such as myotonic dystrophy and facioscapulohumeral muscular dystrophy (FHSD) and a diagnosis can be achieved without the need of a muscle biopsy. Details of clinical features of MDs and other diagnostic techniques are not discussed in the present review. Here we focus on the pattern of protein expression and specific secondary changes in those types of MDs where the analysis of muscle biopsy with commercially available ABs plays a major role in facilitating the diagnosis.

**Table 3:** Primary and secondary protein abnormalities in muscular dystrophies

Disease	Gene(s)	Primary protein defect	Secondary changes
DMD	*DMD*	Dystrophin absent or very reduced with all antibodies	Utrophin overall upregulated Sarcoglycans reduced/absent Dystroglycan reduced/absent nNOS absent

BMD	*DMD*	Dystrophin reduced in size or amount or absent with at least one antibody	Utrophin overall upregulated Sarcoglycans reduced/absent Dystroglycan reduced/absent nNOS may be absent

DMD/BMD carriers	*DMD*	Dystrophin patchy, mosaic pattern with at least one antibody	In fibres without dystrophin Utrophin upregulated Sarcoglycans reduced/absent Dystroglycan reduced/absent nNOS may be absent

EDMD1	*EMD*	Emerin absent	

LGMD1A MFM	*MYOT*	Myotilin cytoplasmic aggregates	Desmin, αB-crystallin, VCP cytoplasmic aggregates

LGMD1B EDMD2 EDMD3	*LMNA*	Lamin A/C normally expressed	

LGMD1C rippling muscle disease hyperCKemia	*CAV3*	Caveolin-3 absent/reduced	Dysferlin reduced at the sarcolemma

LGMD2A	*CAPN3*	Calpain 3 bands may be variably reduced on immunoblot, Iabelling may be absent or reduced on sections	Dysferlin reduced at the sarcolemma

LGMD2B Miyoshi myopathy	*DYSF*	Dysferlin absent or very reduced	Caveolin-3 reduced at the sarcolemma, calpain 3 bands may be reduced

LGMD2C-F	*SGCG SGCA* *SGCB* *SGCD*	Sarcoglycans variably reduced/absent	β-Dystroglycan may be reduced Dystrophin may be reduced nNOS may be absent

LGMD2G	*TCAP*	Telethonin absent	

LGMD2I, K, M-O, DG-pathies	*FKRP POMT1 FKTN POMT2 POMGnT1 LARGE*	Not applicable	Glycosylated α-dystroglycan very reduced/patchy β-dystroglycan may be reduced Laminin α2 may be reduced

MDC1A	*LAMA2*	Laminin α2 completely or partially absent	Laminin α5 overall upregulated α-dystroglycan may be reduced

UCMD and Bethlem myopathy	*COL6A1 COL6A2 COL6A3*	Collagen VI very reduced in UCMD, usually normally expressed in BMD	

MD with lipodystrophy	*PTRF*	PTRF-cavin absent/very reduced	Caveolin-3 reduced at the sarcolemma

MFM	*DES*	Desmin cytoplasmic aggregates	Myotilin, αB-crystallin, VCP cytoplasmic aggregates

MFM	*CRYAB*	αB-Crystallin cytoplasmic aggregates	Myotilin, desmin, VCP cytoplasmic aggregates

IBMPFD	*VCP*	VCP cytoplasmic aggregates	Myotilin, desmin, αB-crystallin cytoplasmic aggregates

BMD = Becker muscular dystrophy; CK = creatine kinase; DG-pathies = dystroglycanopathies; DMD = Duchenne muscular dystrophy; EDMD = Emery-Dreifuss muscular dystrophy; IBMPFD = inclusion body myopathy with Paget's disease and frontotemporal dementia; LGMD = limb girdle muscular dystrophy; MD = muscular dystrophy; MDC = congenital muscular dystrophy; MFM = myofibrillar myopathy; nNOS = neuronal nitric oxide synthase; PTRF = polymerase I and transcript release factor; UCMD = Ulrich congenital muscular dystrophy; VCP = valosin-containing protein.

Genes affected in MDs encode for diverse proteins with various distribution and function within the muscle cell. Direct or functional association between many of these proteins is reflected by the presence of specific secondary abnormalities that are of diagnostic value. In particular, the expression of proteins of the dystrophin glycoprotein complex (DGC) appear to be strictly interrelated, therefore forms of MDs that affect this group of proteins are discussed together in this review.

## Disorders of the dystrophin glycoprotein complex

**Figure 2:**
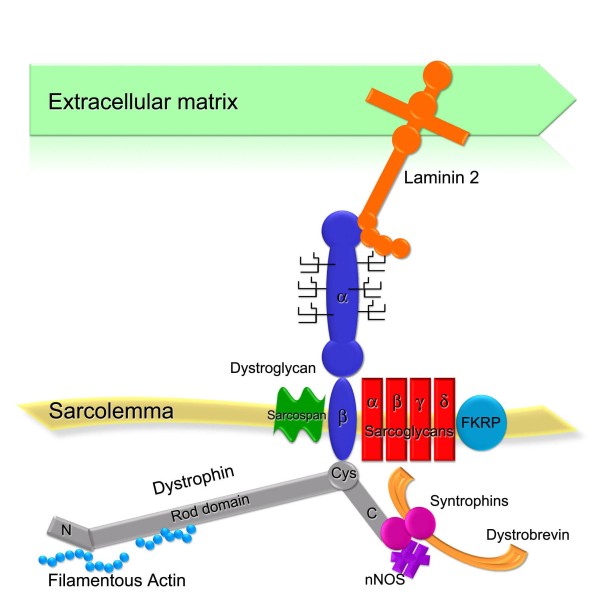
**The dystrophin glycoprotein complex (DGC)**. Schematic representation of the core DGC and associated proteins.

Many of the MD types involve members of the DGC, a group of proteins that links the intracellular cytoskeleton and the extracellular matrix [12-15] (Figure 2). Dystroglycan (α-DG and β-DG) is the transmembrane axis that connects proteins of the extracellular matrix (for example, laminin-2) and dystrophin [12]. The dystrophin molecule has an N-terminal actin-binding domain followed by 24 repeat units (rod domain), a C-terminus that contains a cysteine-rich domain that binds to β-DG [16,17] followed by a region that associates with dystrobrevin, syntrophins and, indirectly, with nNOS [18,19]. In skeletal muscle the DGC also contains four sarcoglycans (α, β, γ and δ-SG) and sarcospan [20], all of which are encoded by different genes. One report also indicates that fukutin-related protein (FKRP) may be an integral component of the DGC [21]. Faults in components or ligands of the DGC complex (as observed in dystrophinopathy, sarcoglycanopathies, dystroglycanopathies and laminin α2 deficiency) cause disruption of the structural link that protects the muscle fibres from damage caused by contraction, and lead to muscular dystrophy [22].

## Dystrophinopathies

Dystrophin is the product of the *DMD *gene, located on the X chromosome. This is a very large gene with a high rate of new mutations with recessive inheritance pattern. Duchenne and Becker muscular dystrophies (DMD and BMD, respectively) are common in male patients, but symptomatic female carriers have also been described [23]. Due to the high incidence of dystrophinopathies compared with other forms of MD, ruling in or out DMD/BMD should always be the first diagnostic step. Deletions of the *DMD *gene account for 65% of cases and can be detected directly by molecular analysis. This test has eliminated the need for muscle biopsy in many cases of 'classic' dystrophinopathies but immunoanalysis still remains an important diagnostic tool [24,25]. Abnormal expression of dystrophin is the hallmark for the diagnosis of DMD/BMD. In general, severe mutations with disruption of the reading frame result in absence of dystrophin and cause DMD, while milder mutations with expression of dystrophin of reduced amount or variable mass cause BMD [26]. Due to the large size of dystrophin it is important to use ABs directed against several sites of the protein, to avoid false-negative results due to deletion of specific epitopes. A panel of ABs directed against N-terminal, C-terminal and rod domain is routinely used in diagnostic laboratories. Western blotting should always be employed in parallel since it offers clues on the type of mutation present. In addition, quantification of the residual amount of dystrophin, if any, is useful for the prognosis of disease severity [27].

**Figure 3:**
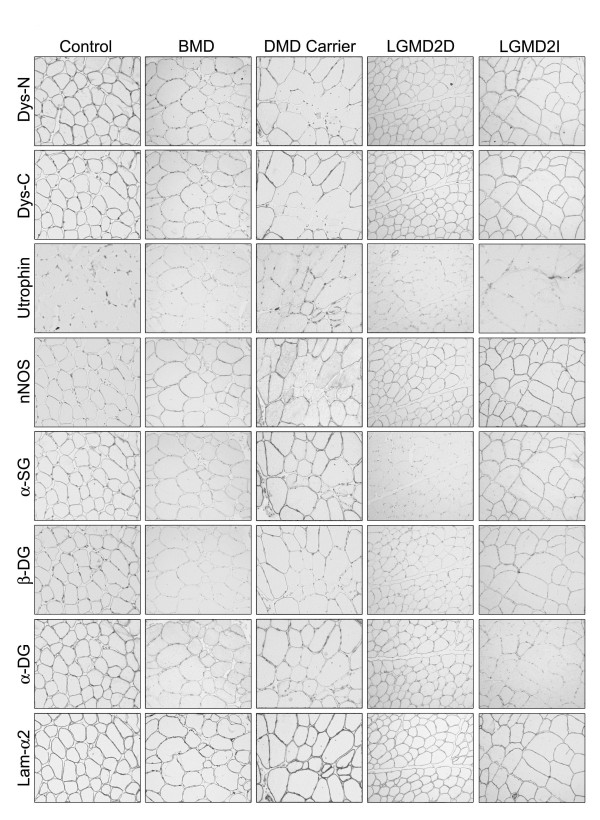
**Primary and secondary protein abnormalities in dystrophin glycoprotein complex (DGC)-related disorders**. A full description is given in the text. BMD = Becker muscular dystrophy; α-DG = α-dystroglycan; β-DG = β-dystroglycan; Dys-C = dystrophin C-terminal; Dys-N = dystrophin N-terminal; lam-α2 = laminin α2; LGMD2D = sarcoglycanopathy with primary defect in the *SGCA *gene; LGMD2I = dystroglycanopathy with primary defect in the *FKRP *gene; α-SG = α-sarcoglycan.

Total absence of dystrophin (Figure 1) or limited labelling on a very small proportion of revertant fibres [28] is specific for the diagnosis of DMD. In contrast, variation in dystrophin expression may be more subtle in patients with BMD. The use of an AB panel for dystrophin is of particular diagnostic value in these cases. Although commercial ABs for dystrophin can detect abnormal protein with the most common in-frame deletions, absence of labelling for an AB indicates deletion of that particular epitope. More frequently, in BMD the intensity of the labelling for one or more AB is variable (Figure 3), but occasionally samples show levels of dystrophin similar to control on sections. Blotting of muscle is especially useful in these cases, however if the abnormality is very subtle, the diagnosis must rely on the assessment of secondary protein changes. Dystrophin expression is often abnormal in carriers of DMD, and its analysis is important in order to exclude differential diagnoses of autosomal MD. Due to a skewed pattern of X-inactivation in muscle, in transverse sections, both positive and negative fibres can be observed in variable proportion, which correlates with the severity of the phenotype [29,30]. Usually in these cases the immunoblot does not show abnormalities since the mutated dystrophin may be masked by the expression of the normal protein [31].

## Secondary protein changes

There is value in including antibodies to other proteins as secondary markers in routine immunohistochemical studies for DMD and, especially, BMD. Utrophin, the dystrophin homolog encoded on chromosome 6, is expressed in normal adult fibres only at the neuromuscular junction, but in DMD/BMD biopsies it is strongly upregulated at the sarcolemma [32]. As utrophin is also expressed in regenerating fibres [33], appropriate markers should be used to distinguish the abnormal from the normal expression (Figure 1). Utrophin is upregulated in most, but not all, cases of BMD [34], therefore it is important to examine the expression of other proteins when the diagnosis is not straightforward (Figure 3). Abnormal expression of dystrophin in DMD, BMD, and manifesting carriers is accompanied by a secondary reduction of other DGC proteins. Reduced labelling for sarcoglycans and α-DG and β-DG is common in dystrophinopathies [13-15,35]. Sarcolemmal nNOS is missing when dystrophin is absent or contains deletions in the mid-rod domain [36], but results have to be interpreted carefully since nNOS expression is very reduced in regenerating and denervated fibres [37].

## Limb girdle muscular dystrophy (LGMD)2C-F (sarcoglycanopathies)

Primary defects responsible for MD have been found in genes encoding for α, β, γ and δ-SG. These cause recessive LGMDs, now often referred to as the sarcoglycanopathies. The SGs act as a complex, such that a defect in any one leads to absence or reduction in expression of the other three SGs (Figure 3) [38]. However this is not a rigorous finding and labelling for all four proteins should be carried out to avoid misdiagnosing cases with selective loss of one SG [39]. The results from ABs to all four SGs are reliable in directing molecular analysis when the immunolabelling for one SG is absent or reduced and the expression of the others is retained [38,40,41]. However in most cases expression of all SGs can be variable and does not allow an accurate prediction of the genotype [42].

## Secondary protein changes

The main clue to differentiate patients with sarcoglycanopathy from BMD or manifesting carriers is a pattern of reduced labelling for SGs in the presence of normal dystrophin (Figure 3). However, reports of sarcoglycanopathy biopsies with abnormal labelling of dystrophin and β-dystroglycan exist [41-43]. The abnormalities in these cases are mild than the defect in SGs expression [44]. In a male, if expression of all SGs and dystrophin is reduced it may be difficult to determine the primary defect without the aid of secondary protein abnormalities. Utrophin is usually not upregulated in LGMD2D-F patients. However, some cases of BMD may not show sarcolemmal utrophin (Figure 3), while occasional sarcoglycanopathies may show utrophin expression [35,45]. Loss of nNOS at the sarcolemma of patients with sarcoglycanopathies has been described [46,47] but the interpretation for diagnostic purposes is subject to the same limitations as for dystrophinopathies.

## Dystroglycanopathies

The gene *DAG1 *encodes the propeptide that is proteolytically cleaved into two non-covalently associated proteins, α-DG and β-DG [48,49]. Primary dystroglycanopathy, due to a defect in *DAG1*, is very rare and has been described in only one patient to date [50]. Secondary dystroglycanopathies are more frequent and are caused by recessive mutations in at least six known or putative glycosyltransferases: FKRP, fukutin (*FCMD*), protein *O*-mannosyl transferase 1 (*POMT1*), protein *O*-mannosyl transferase 2 (*POMT2*), protein *O*-mannosyl β-1,2-*N*-acetylglucosaminyltransferase (*POMGnT1*), and *LARGE *[51,52]. Glycosylation is the crucial modification that modulates the function of α-DG as a receptor for extracellular binding partners [12]. Modifications in these genes result in abnormal glycosylation of α-DG and lead to neuromuscular disorders that range from severe congenital onset with associated brain malformations (Walker-Warburg syndrome, muscle-eye-brain disease, Fukuyama muscular dystrophy), to milder forms of congenital muscular dystrophy (CMD) such as MDC1C and 1D, and to LGMD phenotypes (LGMD2I, 2K, 2M, 2N, and 2O) [53-55]. Irrespective of the gene, all the mutations seem to act in a common pathway and the disease severity is possibly related to the impact of a particular mutation on DG function.

## Secondary protein changes

ABs for most proteins involved are not currently in use for diagnosis. However abnormal labelling for α-DG is common to all the dystroglycanopathies and it is an indicator that the mutation may reside in one of the six genes or in unknown genes in the same pathway. Glycosylation defects are revealed by loss or reduction of immunoreactivity to two ABs, VIA4_1 _and IIH6, which recognise carbohydrate moieties of α-DG (Figure 3) [56]. The extent of α-DG reduction can vary from severe to very subtle and can also be verified by immunoblot. While there is usually a good correlation between reduced α-DG staining and phenotype in patients with mutations in *POMT1*, *POMT2 *and *POMGnT1*, this is not always true in patients with defects in *FKRP *and *FCMD *[57]. Notably, labelling for an AB directed against a core peptide of α-DG (GT20ADG) is usually well preserved on muscle fibres of dystroglycanopathy patients [56]. Consistently, this AB detects hypoglycosylated α-DG with reduced molecular mass but at levels similar to control on immunoblot [56-58]. Variable reduction of laminin α2 labelling can occur, generally more evident on blot [51,59,60]. Laminin α5 is often overexpressed, although the finding is not consistent in all dystroglycanopathy patients (author's observation). β-DG may be mildly reduced on immunohistochemistry, but labelling on blot appears within the normal range [57]. Furthermore, one clinical report suggests that FKRP patients may have reduced expression of other DGC components [61].

## Congenital muscular dystrophy 1A (MDC1A, laminin α2)

Approximately 50% of patients with CMD show deficiency of laminin α2 expression in muscle [60]. Laminin α2, with the laminin chains β1 and γ1, is a component of laminin-2, the major constituent of the basal lamina of muscle fibres. Normal muscle shows uniform sarcolemmal immunolabelling; in cases of MDC1A laminin α2 may be absent or variably reduced [62]. Patients with a partial deficiency usually have a milder phenotype than those with absent or very reduced protein [60]. Laminin α2 is a large protein that on immunoblot appears as 80 and 300 kDa fragments. It is good practice to assess sections of muscle biopsies with ABs to both fragments, particularly to diagnose cases with a partial reduction [5]. Indeed, cases with unremarkable labelling with the 80 kDa AB may show a considerable reduction with the other AB [63]. Laminin α2 is expressed in several tissues that can be used for diagnosis. In skin laminin α2 is localised to the epidermal/dermal junction and to the nerve endings of the arrector pili smooth muscle [64,65]. Prenatal testing can be carried out on chorionic villi from placentas of 8-23 weeks, which express most laminin chains [66]. The absence of laminin α2 is easily observed in these tissues, but cases with partial deficiency may be difficult to identify [64,66,67].

## Secondary protein changes

Upregulation of laminin α5 is observed in cases of total and partial laminin α2 deficiency [62]. This may be especially helpful for the diagnosis of cases with partial reduction, but caution is necessary since laminin α5 is normally present on immature and regenerating fibres. Reduction of α-DG labelling may occur, which generates confusion in cases of partial laminin α2 deficiency [68].

## LGMD1A (myotilin) and myofibrillar myopathies

Myotilin is a sarcomeric protein that is expressed at the Z line of skeletal muscle and is involved in the organisation and stabilisation of thin filaments and the sarcomere [69]. Mutations in the *MYOT *gene with LGMD1A phenotype have been identified in only two unrelated families [70,71], but the gene is also mutated in other forms of muscle disease such as myofibrillar myopathy (MFM), spheroid body myopathy, and late onset distal myopathy [72-74]. Signs of myotilinopathy include granular or hyaline accumulations that stain dark blue or red with Gomori trichrome (cytoplasmic bodies), vacuoles frequently rimmed by basophilic or red material with haematoxylin and eosin and Gomori trichrome, respectively, and streaming Z lines [72]. These structural abnormalities are also common features of the large group of MFMs [75], caused by mutations not only in *MYOT *but also in a number of other genes. MFM genes identified so far encode for desmin, αB-crystallin, Z band alternatively spliced PDZ-motif-containing protein (ZASP), filamin C, four-and-a-half LIM domain 1 (FHL1), BCL2-associated athanogene 3 (BAG3), and plectin (for review see [76]). This class of dominantly inherited diseases is difficult to differentiate based on histology only and clinical features are often better indicators of where the primary genetic defect may lie. Some histological findings can facilitate the diagnosis, for example spheroid bodies in Gomori trichrome stain may indicate a myotilinopathy or sarcoplasmic reducing bodies positive for menadione-NBT indicate FHL1opathy [76,77]. Antibodies directed against desmin, αB-crystallin and myotilin are good diagnostic tools to detect protein accumulation in MFM. However the number of affected fibres can be very variable, with some samples showing a large abundance and others only displaying a few abnormal fibres. Therefore protein tests may be unspecific or normal in genetically confirmed MFMs [76]. Furthermore, it must be kept in mind that minicores, central cores, and target fibres also show increased reactivity with these antibodies [76]. Accumulations of other sarcolemmal proteins such as dystrophin, SGs and caveolin-3 may also be seen in MFM [75]. Similar findings may be observed in biopsies from patients with valosin-containing protein (VCP)-related inclusion body myopathy associated with Paget's disease of bone and frontotemporal dementia which, therefore, should not be excluded as a differential diagnosis [78]. In summary, histology and immunoanalysis in the context of clinical data are useful to achieve a generic diagnosis for MFM, but immunolabelling is rarely conclusive in identifying the affected gene.

## LGMD1C (caveolin-3)

**Figure 4:**
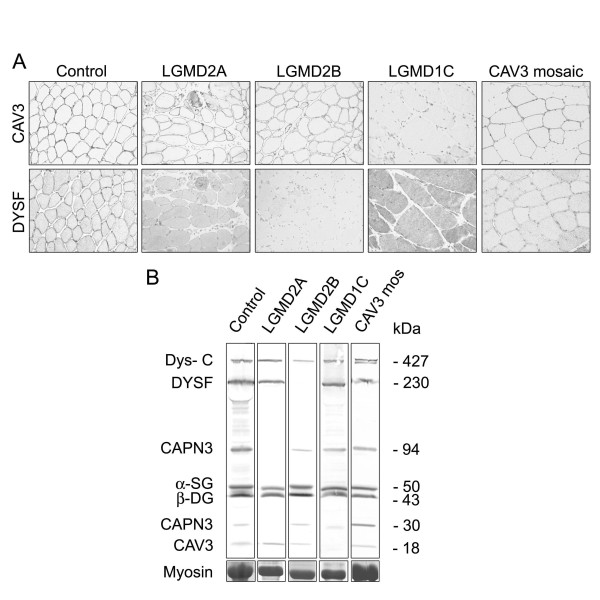
**Comparative expression of dysferlin, caveolin 3 and calpain 3**. Expression of these proteins is primarily or secondarily affected in patients with defects in the caveolin 3 (*CAV3*) gene (limb girdle muscular dystrophy (LGMD)1C), the dysferlin (*DYSF*) gene (LGMD2B) and the calpain 3 (*CAPN3*) gene (LGMD2A). In a patient with mosaic expression of caveolin 3, fibres with loss of the protein also show reduced sarcolemmal labelling for dysferlin **(a)**. **(b) **Immunoblot is a more reliable technique for diagnosis of LGMD2A and 2B. β-DG = β-dystroglycan; Dys-C = dystrophin C-terminal; α-SG = α-sarcoglycan.

Mutations in the *CAV3 *gene cause LGMD1C [79,80]. The muscle biopsies show a severe reduction of caveolin-3 at the sarcolemma. Caveolin-3 is localised at small invaginations of the plasma membrane (caveolae) and interacts with β-DG, but it is not an integral part of the DGC [81]. Mutations in *CAV3 *have also been found in hyperCKemia, distal myopathy, and rippling muscle disease, with many patients showing an overlap of the phenotypes. Although these disorders are mainly inherited in an autosomal dominant fashion, occasional autosomal recessive cases have been reported [82]. A mosaic pattern of reduction of caveolin-3 at the sarcolemma is detected in patients with immune-mediated rippling muscle disease (Figure 4). In these patients caveolin-3 may not be significantly reduced on blots [83]. More recently, secondary reduction of caveolin-3 at the sarcolemma has been described in patients with mutations in the gene encoding for polymerase I and transcript release factor (PTRF)-cavin, a caveolar-associated protein that plays a role in the formation of caveolae and the stabilisation of caveolins [84,85]. Abnormal localisation of dysferlin is often seen in muscle from patients with LGMD1C, indicating a structural or functional interaction between these proteins [86]. In the absence of caveolin-3, dysferlin accumulates in the cytoplasm and displays an irregular sarcolemmal distribution (Figure 4).

## LGMD2A (calpain 3)

Calpain 3 (CAPN3), a muscle specific calcium-activated neutral protease, is the defective protein in LGMD2A [87]. Several cytoskeletal proteins are substrates for CAPN3, but this protein also displays autolytic activity and it is cleaved into a N-terminal fragment of 30 kDa and C-terminal fragments of 60 to 45 kDa [88,89]. Two ABs are mainly used for the detection of CAPN3 (Table 1). Until recently [90,91], it was thought that these only work on immunoblot where they produce characteristic patterns of bands: full length CAPN3 (94 kDa), plus the additional degradation fragments (Figure 4 and data not shown) [92]. Immunocytochemical studies of CAPN3 are limited but promising, showing intracellular labelling with both ABs, which is absent in most LGMD2A patients [90,91]. Dysferlin membrane labelling may be secondarily reduced (Figure 4) [93].

Protein-based diagnosis for LGMD2A is not always fully reliable. Expression of full-length protein and degradation fragments has to be expertly evaluated to establish whether a defect is genuine or secondary. Indeed numerous factors influence the expression and detection of CAPN3. Secondary deficiencies of CAPN3 have been described in several muscular dystrophies, including LGMD2B and LGMD2J [94,95]. Moreover, CAPN3 reduction is often observed in many dystrophic biopsies, perhaps as a consequence of rapid degradation and/or biopsy processing conditions [96]. In contrast, normal CAPN3 expression may be seen in patients with confirmed molecular diagnosis of LGMD2A [97]. Therefore data on CAPN3 expression must be interpreted by taking into account issues related to protein degradation and abnormalities of other LGMD proteins. For patients with a strong clinical suspicion for LGMD2A and normal CAPN3 expression the diagnosis relies on genetic analysis, but there is also value in using functional *in vitro *assays for the autolytic activity of CAPN3 [98].

## Dysferlinopathies

Dysferlin is the product of the gene responsible for LGMD2B, Miyoshi myopathy and distal anterior compartment myopathy [99-101]. This protein is widely distributed, although it predominates in striated muscle where it localises to the sarcolemma and intracellular vesicles [102,103]. Primary defects in the *DYSF *gene lead to severely reduced protein expression and subsarcolemmal accumulation of vesicles [104]. CAPN3 and caveolin-3 are often secondarily reduced in LGMD2B (Figure 4) [105,106]. Secondary sarcolemmal reduction of dysferlin is often seen in muscle biopsies of patients with many other forms of muscular dystrophy but in all these cases the band for dysferlin is normally detected on immunoblot [86,104,107,108]. In contrast LGMD2B and Miyoshi myopathy patients typically show complete or partial absence of the dysferlin band on immunoblot of skeletal muscle (Figure 4b) or blood monocytes [109,110]. Some heterozygous carriers of LGMD2B also display reduced dysferlin levels, as well as variable muscle histopathological changes [111].

## LGMD2G (telethonin)

LGMD2G is a rare condition described mainly in the Brazilian population [112,113] and it is associated with mutations in the gene for telethonin. Telethonin is a sarcomeric protein expressed at the Z line in skeletal and cardiac muscle, where it interacts with the C-terminal domain of titin [69,114]. Examination of muscle biopsies from affected individuals shows absence of telethonin and maintenance of the sarcomeric structure. All the other proteins involved in muscular dystrophies are normally expressed [114].

## Emery-Dreifuss muscular dystrophy (EDMD)

Mutations in a number of genes are responsible for EDMD. Interestingly, all genes involved encode for proteins of the nuclear membrane. Both X-linked and autosomal forms have been described. EDMD1 is caused by mutations in the *EMD *gene on the X chromosome that encodes for emerin [115]. Mutations occur throughout the gene and result in absence or mislocalisation of the protein [116,117]. In female carriers a variable number of emerin-positive and emerin-negative cells can be detected [118]. Emerin is ubiquitously expressed, and many tissues can be used as an alternative to muscle for protein studies. In particular skin and cells from buccal smears are frequently tested for diagnosis of X-linked EDMD patients and female carriers [119-121].

EDMD2 is due to mutations in the *LMNA *gene that encodes for lamins A and C, intermediate filaments of the inner nuclear membrane in almost all cells. Mutations in *LMNA *can cause several different phenotypes including LGMD1B (for review see [122]). Inheritance is autosomal dominant, but very rare autosomal recessive cases (EDMD3) have been described [123]. Immunoanalysis in this type of disorder is not informative as no difference in the expression of lamin A/C in skeletal muscle is detected due to the expression of the normal allele.

New mutations have been found in the synaptic nuclear envelope protein 1 (*SYNE1*) and in the synaptic nuclear envelope protein 2 (*SYNE2*) genes in two patients in two families, also called Nesprin-1 and Nesprin-2, but too few cases have been identified to know if immunolabelling has a role in diagnosis [124]. Mutations in the *FHL1 *gene have also been described in association with X-linked EDMD [125].

## Ullrich congenital muscular dystrophy (UCMD) and Bethlem myopathy (collagen VI)

Collagen VI is a protein of the extracellular matrix, which consists of three *α *chains, α1(VI) α2(VI) and α3(VI), encoded by the *COL6A1*, *COL6A2 *and *COL6A3 *genes, respectively. Mutations in all three genes have been reported in UCMD families [126-128] and in the milder Bethlem myopathy [129,130]. Collagen VI is present in most connective tissues and, in skeletal muscle, localises in the basement membrane, endomysium and perimysium. In the muscle of UCMD patients a spectrum of collagen VI anomalies can be found. The protein can be completely absent or the changes can be difficult to detect, with only absence at the basal lamina [127,131]. The subtle reduction makes it important to evaluate the results by double labelling with another antibody such as perlecan to verify the integrity of the basal lamina [5]. It must be noted that normal or nearly normal collagen VI immunolabelling does not exclude a diagnosis of UCMD [132]. In Bethlem myopathy, collagen VI immunolabelling of muscle is usually normal or shows very subtle alterations [133].

Several reports have described an absence or a reduction in the amount of secreted collagen VI in fibroblast cultures from UCMD patients [128,134]. This technique has a much higher predictive value of *COL6A *mutations in BM patients, since it allows detection of even very subtle alterations in collagen VI expression and secretion [133].

## Conclusions

Protein analysis is the most valuable and dependable way of improving the efficiency of genetic testing in many types of MDs. Diagnosis is achieved in a significant proportion of patients when informative clinical data and immunoanalysis results are available to guide the molecular analysis [135]. The muscle biopsy is a time and cost-effective test for many muscle disorders with ambiguous presentation in part because of the limited capacity to screen for large and numerous genes simultaneously. When the primary genetic modification is unknown, immunoanalysis may be very useful to identify primary or secondary changes that highlight direct or functional protein interactions and guide the search for candidate genes. As molecular technologies are rapidly evolving, it is likely that in the near future low cost sequence for all MDs genes at once will be available. These advances will transform the diagnostic pathway for MDs and the diagnostic role of muscle biopsy may become less central. However, despite the availability of high throughput genetic tests, studies of protein expression in muscle biopsies will still be necessary for the diagnosis of MD. In fact, the effect of novel mutations is not always predictable and disruption or preservation of protein synthesis needs to be confirmed in the muscle. In addition protein analysis has important prognostic value, as seen for example in dystrophinopathies, where the residual amount of dystrophin correlates better with the phenotype than the genetic prediction [136]. Furthermore, tests on muscle biopsy remain crucial for evaluating the success of applied research and clinical trials such as gene therapies that aim to restore the missing protein.

## Competing interests

The author declares that they have no competing interests.
